# Dendritic Cell-Associated miRNAs Are Modulated via Chromatin Remodeling in Response to Different Environments

**DOI:** 10.1371/journal.pone.0090231

**Published:** 2014-04-03

**Authors:** Shiyue Mei, Yuanhang Liu, Yue Bao, Yuan Zhang, Siping Min, Yifei Liu, Yun Huang, Xidi Yuan, Yue Feng, Jiandang Shi, Rongcun Yang

**Affiliations:** 1 Department of Immunology, Nankai University School of Medicine, Nankai University, Tianjin, P. R. China; 2 Key Laboratory of Bioactive Materials, Ministry of Education, Tianjin, P. R. China; 3 Department of Biochemistry, College of Life Science, Nankai University, Tianjin, P. R. China; Indian Institute of Science, Bangalore, India

## Abstract

**Introduction:**

Epigenetic modification plays a critical role in regulating gene expression. To understand how epigenetic modification alters miRNA expression in monocyte-derived dendritic cells (moDCs) in different environments, we analyzed the connections between H3K4me3 and H3K27me3 modification and the expression of miRNAs in LPS- and TGF-β-conditioned moDCs.

**Results:**

In moDCs, H3K4me3 modification was strongly associated with the expression of activating miRNAs, whereas H3K27me3 was related to repressive miRNAs. The regulation of miRNA expression by H3K4me3 and H3K27me3 was further confirmed by silencing or inhibiting methyltransferases or methylation-associated factors in LPS- and TGF-β-conditioned moDCs. siRNAs targeting H3K4me3-associated mixed lineage leukemia (MLL) and retinoblastoma binding protein 5 (RBBP5) reduced H3K4me3 enrichment and downregulated miRNA expression; conversely, silencing H3K27me3-associated enhancer of zeste homolog 2 (EZH2) and embryonic ectoderm development (EED) genes upregulated the DC-associated miRNAs. However, LPS-mediated miRNAs were often associated with H3K4me3 redistribution from the transcription start site (TSS) to the miRNA-coding region. Silencing LPS-associated NF-κB p65 and CBP/p300 not only inhibited H3K4m3 redistribution but also reduced miRNA expression. LPS-upregulated RBBP4 and RBBP7, which are involved in chromatin remodeling, also affected the redistribution of H3K4me3 and reduced the expression of miRNAs.

**Conclusion:**

In LPS- and TGF-β-conditioned moDCs, miRNAs may be modulated not only by H3K4m3 and H3K27me3 modification but also by redistribution of H3K4me3 around the transcriptional start site of miRNAs. Thus, H3K4me3 and H3K27me3 epigenetic modification may play an important role in regulating DC differentiation and function in the presence of tumor or inflammatory environments.

## Introduction

MicroRNAs (miRNAs) are short non-coding RNAs (21–23 nt) that are present in multiple organisms and are often evolutionarily conserved [1]. miRNAs have emerged as key post-transcriptional regulators of gene expression in mammals [2], with each miRNA targeting dozens or possibly hundreds of mRNAs. More than 1000 miRNAs are encoded in the mammalian genome. These miRNAs are derived from a complex series of processing steps [3], [4]. The precursors of miRNAs, known as pri-miRNAs, similarly possess a 5′ cap structure and a 3′ polyA tail [5]. Most pri-miRNAs are transcribed similarly to protein-coding genes [4].

Dendritic cells (DCs) are extremely versatile antigen-presenting cells involved in both innate and adaptive immunity and in tolerance maintenance. The diverse functions of DCs in immune regulation depend not only on the heterogeneity of DC subsets but also on their functional plasticity [6]–[9]. Under different stimuli, DCs may acquire distinct specialized and polarized functional properties [10], [11]. DCs express a variety of pathogen recognition receptors, such as Toll-like receptors (TLRs), to initiate DC maturation and migration to the regional lymph nodes [7], [12]. Upon exposure to TGF-β, DCs become tolerogenic. In this way, they suppress T-cell alloreactivity [13] and induce Th2 or T regulatory responses [7], [12]. More than 100 miRNAs are selectively expressed in adaptive and innate immune cells [14]. The expression of miRNAs is markedly different in conditioned DCs and differs among subsets of DCs [15]. Because DC function depends on the cellular concentration of miRNA, the regulation of miRNA expression and abundance during ontogeny is not completely clear.

Although multiple mechanisms are involved in regulating miRNA expression in different cell types, histone methylation and demethylation are important regulatory mechanisms in cellular differentiation. Trimethylated histone H3K4 (H3K4me3) marks transcriptional start sites (TSSs) in virtually all active genes [16], whereas trimethylation of histone H3K27 (H3K27me3) is related to gene repression [17]. According to recent studies, H3K4me3 modification depends on the MLL (myeloid/lymphoid or mixed lineage leukemia) and RBBP5 (retinoblastoma Binding Protein 5) complex, while the EZH2 (enhancer of zeste homolog 2) enzyme and EED (embryonic ectoderm development) protein play an important role in H3K27me3 methylation [18]–[20]. Other key components of the chromatin remodeling process are RBBP4 (retinoblastoma Binding Protein 4) and RBBP7 (retinoblastoma Binding Protein 7) [21]. Epigenetic modification has been reported to affect the differentiation and function of immune cells [22]–[28]. Global mapping of H4K4me3 and H3K27me3 has revealed the specificity and plasticity of lineage fate determination in differentiating CD4^+^ T cells [22], in memory CD8^+^ T cells [23] and in human embryonic stem cells [24]. In LPS-conditioned macrophages, gene-specific regulation occurs at the chromatin level, and involves nucleosome remodeling and covalent histone modifications [25]. The inhibition of methyltransferases, methylation-associated factors or histone deacetylases (HDAC) also affects the expression of multiple genes in DCs [26], [27]. The characteristics of H3K4me3 and H3K27me3 histone modification have been described in human moDCs [28] and in human TGF-β and LPS-conditioned moDCs [27]. In this paper, we analyze the effects of H3K4me3 and H3K27me3 on miRNA expression in LPS- and TGF-β-conditioned moDCs. We found that both H3K4me3 or H3K27me3 enrichment and the redistribution of these epigenetic marks regulate miRNA expression during the transition from immature to activated moDCs (LPS-conditioned moDCs) and tolerized moDCs (TGF-β-conditioned moDCs).

## Materials and Methods

### Preparation of human monocyte-derived dendritic cells

moDCs were prepared from the buffy coats of healthy donor samples obtained from the blood bank of Tianjin, P. R. China, as previously described [48]. The study was approved by the Institute Research Ethics Committee at Nankai University (Permit No: 200828). Briefly, peripheral blood mononuclear cells (PBMCs) were separated by Ficoll-Paque gradient centrifugation, and CD14^+^ cells were purified using antibody-coated microbeads and magnetic separation. Selected CD14^+^ cells (2×10^6^/ml) were cultured for 5 days in the presence of GM-CSF (500 U/ml) and IL-4 (500 U/ml) (R&D, USA) to generate immature moDCs. The immature moDCs were matured in the presence of lipopolysaccharides (LPS) (100 ng/ml, Invivogen, USA) or rendered tolerant in the presence of TGF-β (10 ng/ml, R&D, USA). Incubation time for maturation or tolerance acquisition was 24 hrs.

### miRNA array

Expression levels of miRNAs in moDCs, LPS-conditioned moDCs and TGF-β-conditioned moDCs were analyzed using Exiqon microRNA arrays (Denmark). Total RNA was isolated using the TRIZOL® Reagent (Invitrogen Life Technologies). The concentration and purity of the RNAs were determined using the NanoDrop® ND-1000. The miRNAs were labeled using the miRCURY™ Array Power Labeling kit (Exiqon). miRNA array hybridization was performed, and unbound miRNA labels were washed away using the miRCURY™ Array wash buffer kit (Exiqon). The arrays were scanned on an Axon 4000B scanner (Molecular Devices), and the signal intensity was determined using GenePix Pro 6.0 software (Molecular Devices). miRNAs with a 1.2-fold increase or decrease in expression were regarded as differential expression.

### Flow cytometric analyses

For flow cytometric analyses, cells were collected in ice-cold PBS and incubated with the indicated PE- or FITC-labeled antibodies. In each analysis, isotype-matched control mAb was used as a negative control. The phenotypes of human moDCs were analyzed using PE- or FITC- labeled anti-CD14 (61D3), anti-CD83 (HB15e), anti-CD11c (3.9), anti-CD86 (IT2.2), anti-CD80 (2D10.4), anti-CD40 (HB14), and anti-CD11b (ICRF44), purchased from PharMingen, USA.

### RNA isolation and quantitative real-time PCR

Total RNA was extracted using the TRIzol reagent (Invitrogen, USA), and reverse transcription was carried out with Superscript III (Invitrogen, USA), following the manufacturer's protocols. Expression of genes and mature miRNAs was assayed by quantitative real-time PCR (qRT-PCR) using the Quantitect SYBR PCR kit with a specific primer set recommended by the manufacturer (Qiagen, USA). As an endogenous control for gene expression, the GAPDH mRNA in gene samples and U6 in mature miRNA samples were amplified during each sample experiment. Fold changes were calculated using the ΔΔC_t_ method, following the manufacturer protocol (Applied Biosystems, USA). All reactions were run in triplicate and the primer sequences were listed in Table S1.

### ChIP, ChIP-Seq and bioinformatics

ChIP (chromatin immunoprecipitation) and ChIP-seq (chromatin immunoprecipitation followed by high throughput sequencing) were performed by the Research & Cooperation Division of BGI-Shengzhen, China, as previously described [27]. The specificity of the immunoprecipitation was confirmed by qRT-PCR analysis of known genes; namely, GAPDH (which encodes transcriptionally active euchromatin), human MYOD1 (encoding transcriptionally inactive euchromatin) and human SAT2 (encoding heterochromatin). The primers were obtained from SABiosciences. DNA fragments of approximately 200 bp (mononucleosomal DNA+adaptor) were selectively recovered from 2% agarose gels for further clustering and sequencing with the Solexa/Illumina 1G Genome Analyzer.

All bioinformatics analyses were based on previously described methods [27].

### ChIP-qRT-PCR assays

The cells were plated into 15-cm dishes (density = 5×10^6^ cells per dish) and treated with different siRNAs. After 24 hrs of treatment, ChIP assays were carried out following the protocols in the ChIP assay kit (UPSTATE, USA). For each ChIP assay, anti-human H3K4me3 and anti-human H3K27me3 antibodies or immunoglobulin control were used. ChIP enriched DNA and input DNA were subjected to qRT-PCR analysis with Maxima SYBR Green/ROX qPCR Master Mix (Fermentas Inc.). ChIP enrichment was assessed relative to the input DNA in specific genomic regions. The primers sets were listed in Table S1.

### siRNA experiments

siRNAs for MLL, RBBP4, RBBP5, RBBP7, EZH2, EED, NF-κB p65, NF-κB p50, CPB(CREB binding protein)/p300, HDAC1 and control oligos (mock siRNAs) were purchased from Guangzhou Ribobio, China and were transfected into cells with Entranster™-R, as recommended by the manufacturer (Engreen Biosystem, China). The siRNAs (whose sequences are listed in Table S1) were designed and synthesized by Guangzhou Ribobio, China. The non-silencing mock siRNAs contained random nucleotides (ACTATCTAAGTTACTACCC). To determine the effects of MLL, RBBP4, RBBP5, RBBP7, EZH2, EED, p65, p50, p300 and HDAC1 knockdown on miRNA expression, the cells were transfected with different siRNAs, and miRNA expression was analyzed 24 hrs later. The transcriptional levels of the genes were detected by qRT-PCR. To determine the effects of MLL, RBBP4, RBBP5, RBBP7, EZH2, EED, p65, p50, p300 and HDAC1 knockdown on H3K4me3 enrichment, the cells were transfected with different siRNAs and assayed by ChIP-PCR after 24 hrs. The primers used in these experiments were listed in Table S1.

### ELISA

TNF-α (Jingmei Biotech Co. Ltd, China) was quantified using commercial sandwich ELISA kits and a SpectraMax 190 (Molecular Devices, USA) microtiter plate reader. Cytokine levels from two to three titrations were quantified using standard curves, and expressed in pg/ml.

### Statistical analysis

Data are presented as the mean ±SD (standard deviation). Statistical comparisons were performed with Student's *t*-test. A two-sided *p*-value less than 0.05 was considered statistically significant.

## Results

### H3K4me3 modification is associated with active miRNAs, whereas repressive miRNAs are related to H3K27me3 in moDCs

**Figure 1:**
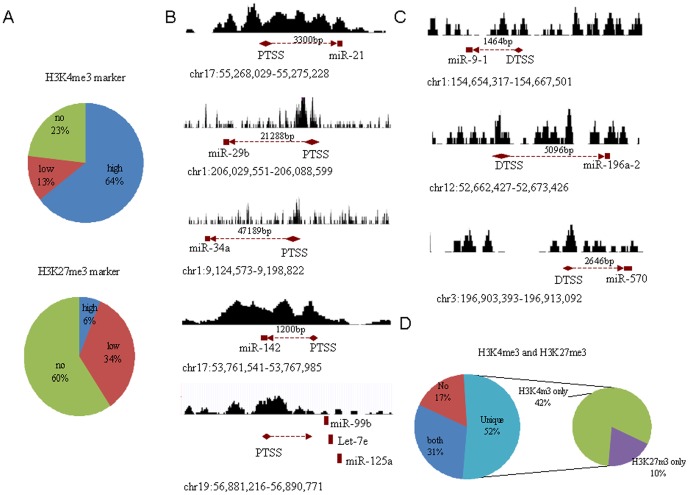
In moDCs, active and repressive miRNAs are associated with H3K4me3 and H3K27me3 modification, respectively. (**A** and **B**) Proportion of moDCs-associated miRNAs with H3K4me3 or H3K27me3 modification. High, miRNAs with remarkable H3K4me3 or H3K27me3 modification; low, miRNAs with less H3K4me3 or H3K27me3 modification; no, miRNAs without H3K4me3 or H3K27me3 modification. (**C**) Repressive miRNAs with H3K27me3 modification. (**D**) Proportion of moDCs associated miRNAs with both H3K4me3 and H3K27me3 modification (Both). MoDCs were generated and visualized with anti-CD14, anti-CD83, anti-CD11c, anti-CD86, anti-CD80, anti-CD40 and anti-CD11b [27]. The gene structure was downloaded from the UCSC Genome Brower. Enriched regions were found in the UCSC Genome Browser (http://genome.ucsc.edu.). Histone modification peaks were detected by CHIPOTle. miRNA positions were predicted by Ensembl. The definitive and putative miRNA TSSs were inferred from http://mirstart.mbc.nctu.edu.tw/browse.php. PTSS, putative transcription start site; DTSS, definitive transcription start site.

In a previous study, we revealed the epigenetic features presented during moDC differentiation, and generated global maps of H3K4me3 and H3K27me3 modification in three cell types (moDCs, LPS-conditioned moDCs and TGF-β-conditioned moDCs) using the ChIP-Seq approach [27]. Here, we investigated the effects of H3K4me3 and H3K27me3 modification on miRNA expression in the same cell types. Since miRNA expression profiles differ markedly in different organs, tissues and cell lineages [29], we first analyzed the expression of miRNAs in moDCs using miRNA arrays. Data revealed that 859 miRNAs could be detected in moDCs, LPS- and TGF-β-conditioned moDCs. 93 of 859 miRNAs (11%) in LPS-conditioned moDC and 23 (3%) in TGF-β-conditioned moDCs were upregulated; whereas 42 of 859 miRNAs (5%) in LPS-conditioned moDC and 131 (16%) in TGF-β-conditioned moDCs were down-regulated (Fig. S2 and Table S2). The transcription start sites (TSSs) of the miRNAs are listed at http://mirstart.mbc.nctu.edu.tw/browse.php. The putative TSSs (PTSSs) were predicted from published experimental evidence, including cap analysis of gene expression (CAGE) tags, TSS Seq tags and H3K4me3 enrichment [30]. 128 of 209 miRNAs (64%), which could be detected in untreated moDCs underwent remarkable H3K4me3 modification (representative samples were shown in Fig. 1B), while 13 miRNAs (6%) showed significant H3K27me3 modification around their TSS (for representative samples, see Fig. 1C). 64 miRNAs (31%) had modifications of both H3K4me3 and H3K27me3 (Fig. 1D). Importantly, most of the 128 miRNAs with H3K4me3 modification (Fig. 1B and Table S3), including miRNA-146, miR-155, Let-7a, Let-7b, miR-20, miR-23b, miR29b, miR-34a, miR-99b and miR-148/152, belonged to active miRNA (31). These miRNAs play critical roles in regulating the maturation, differentiation and functions of DCs [15], [31]. miRNAs that are generally repressive in DCs were significantly modified by H3K27me3, such as miR-9-1, miR-196a and miR-570 (Fig. 1C). Thus, in moDCs, H3K4me3 modification is associated with active miRNAs, whereas repressive miRNAs are related to H3K27me3.

### Silencing of epigenetic factors affects the enrichment of H3K4me3 and expression of miRNAs

**Figure 2:**
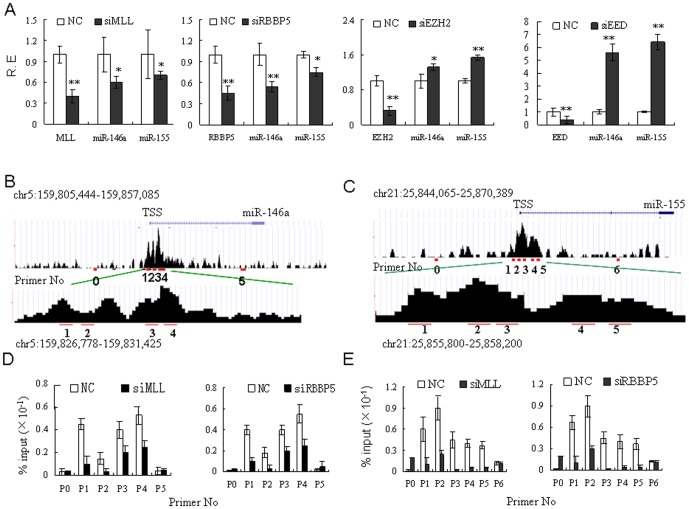
Silencing of epigenetic factors affects miRNA expression and H3K4me3enrichment. (**A**) qRT-PCR analysis of miR-146a and miR-155 in moDCs transfected with various proteins. MoDCs were respectively transfected by siRNAs against MLL (siMLL), RBBP5 (siRBBP5), EZH2 (siEZH2), EED (siEED) or by mock siRNA as negative control (NC). After 48 hrs, total RNA was extracted, and miR-146a and miR-155 expression was analyzed. (**B** and **C**) H3K4me3 and H3K27me3 modifications at the TSS of miR-146a and miR-155. Significant histone modifications peaks were identified by CHIPOTle. The miRNA positions are predicted by Ensembl. The positions of primers used in ChIP-PCR are labeled in red. TSS, transcription start site. (**D** and **E**) ChIP-PCR analysis of H3K4me3 modification around the TSSs of miR-146a and miR-155. MoDCs were transfected with siMLL, siRBBP5 or NC. ChIP was performed with an anti-H3K4me3 antibody, and H3K4me3 enrichments were analyzed by qRT-PCR. Enrichments (relative to the input DNA in specific genomic regions) were assessed by ChIP. The primer sets are listed in Table S1. The qRT-PCR data are representative of three healthy donors. The arrow points in the direction of gene transcription. R.E, relative expression. *, P<0.05; **, P<0.01.

According to previous studies, MLL and RBBP5 mediate H3K4me3 methylation [32], [33] while EZH2 and EED influence H3K27me3 methylation [18]–[20]. We next investigated the effects of these H3K4me3- and H3K27me3-associated factors on the expression of miR-146a and miR-155 by ChIP-PCR. We also analysed H3K4me3 accumulation at the miR-146a and miR-155 promoters. miR-155 and miR-146a are known to mediate the function and differentiation of DCs [15]. Expression of miR-155 and miR-146a is upregulated during maturation of numerous mouse and human DCs in response to various TLRs [34]. In the present study, MoDCs were transfected with siRNAs against MLL, RBBP5, EZH2 or EED (Fig. 2A). As shown in Fig. 2A and Fig. S1, silencing MLL and RBBP5 heavily repressed miR-146a and miR-155expression, whereas silencing EZH2 and EED upregulated it (relative to the control; P<0.01). ChIP supplied with antibodies for H3K4me3, followed by qRT-PCR with primers flanking the H3K4me3 around the miR-146a and miR-155 TSSs (Fig. 2B and C) showed that by silencing MLL and RBBP5, H3K4me3 accumulation was inhibited at both the miR-146a and miR-155 promoters (Fig. 2D and E). However, the effect of EZH2 and EED on H3K27me3 accumulation was less clear because of diffusion in the H3K27me3 enrichment peaks. The expression of miRNAs in moDCs was also modulated by methyltransferase inhibitor AdOx, histone-lysine N-methyltransferases EZH1 and EHMT2, and chromobox homolog 5 (CBX5) (data not shown). In addition, by silencing MLL, RBBP5, EZH1, CBX5 and EHMT2, cytokine production and expression of co-stimulatory molecules was suppressed (Fig. S1 and [27]). All of these data suggest that H3K4me3 and H3K27me3 modifications are involved in miRNAs regulation in moDCs.

Notably, some active miRNAs in human moDCs, such as miR-10a, miR-132, miR-125b, miR-212 and miR-511 [28], have been shown to lose or reduce their H3K4me3 mark independently of their gene expression (Table S3). Some repressive miRNAs such as miR-181b-1, miR-130, miR-101-1 and miR-101-2 were similarly independent of H3K27me3 modification (Table S3). These results suggest that both H3K4m3 and H3K27me3 marks are unstable and prone to either loss of differentiation state or gain of another permissive mark, such as H3K9me3 [35].

### TGF-β-associated miRNAs are modulated by H3K4me3 and H3K27me3 modifications

**Figure 3:**
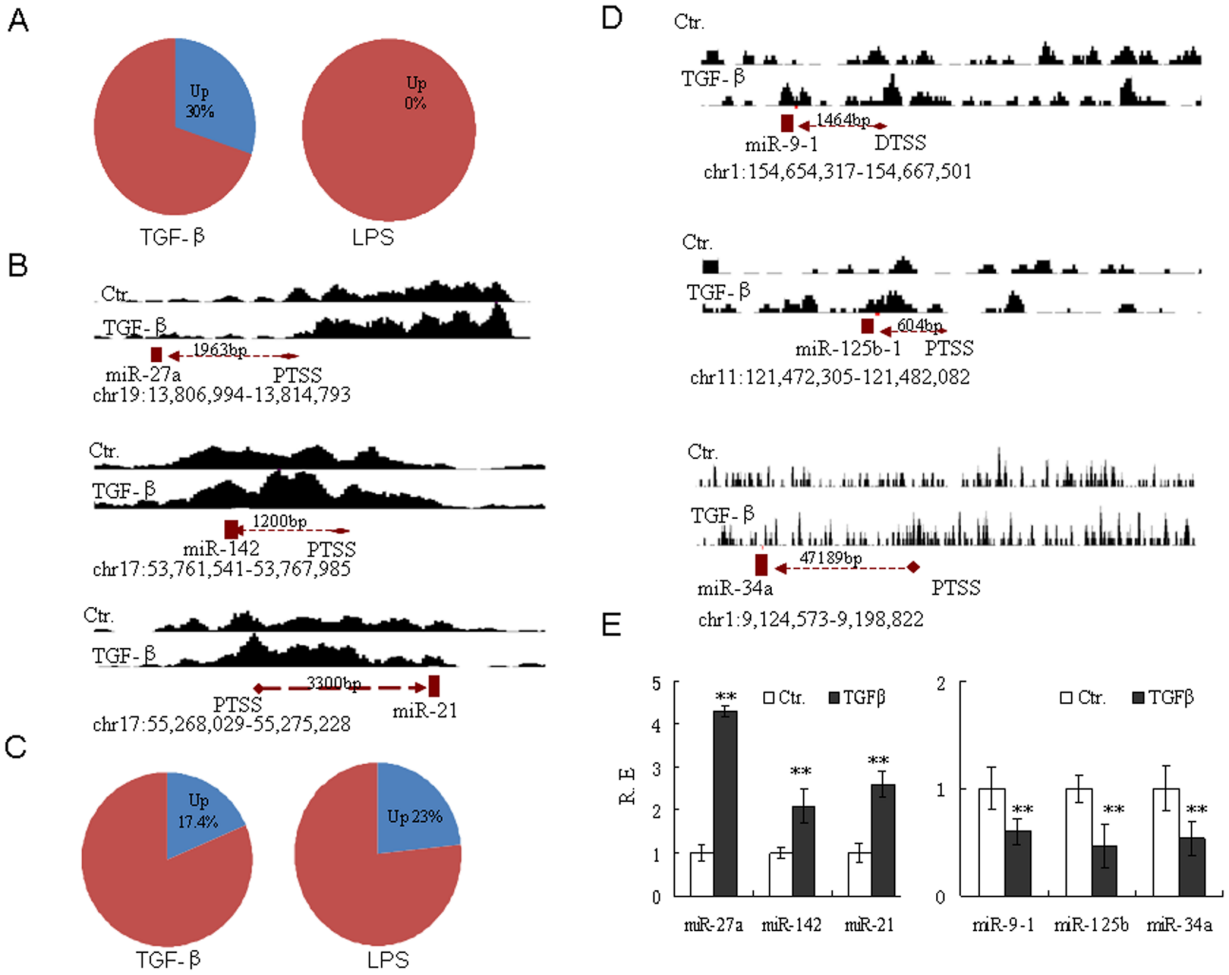
H3K4me3 and H3K4me27 modifications are involved in regulating miRNA expression in TGF-β-conditioned moDCs. (**A**) Proportion of miRNAs with increased H3K4me3 enrichment (Up) in TGF-β-conditioned moDCs (TGF-β) or in LPS-conditioned moDCs (LPS). (**B**) Increased H3K4me3 enrichment (Up) appears around the TSS in TGF-β or LPS-up-regulated miRNAs. (**C**) Proportion of miRNAs with increased H3K27me3 enrichment (Up) in TGF-β-conditioned moDCs (TGF-β) or in LPS-conditioned moDCs (LPS). (**D**) Increased H3K27me3 enrichment appears around the TSS in TGF-β or LPS down-regulated miRNAs. (**E**) Expression of increased H3K4me3 or H3K27me3 enrichment miRNAs in TGF-β-conditioned moDCs. Total RNAs were extracted from TGF-β-conditioned moDCs. To prepare them for ChIP-Seq analysis, the generated moDCs were exposed to TGF-β (10 ng/ml) for 24 hrs. The gene structure was downloaded from the UCSC Genome Brower. Enriched regions were found in the UCSC Genome Browser (http://genome.ucsc.edu.). Histone modification peaks were detected by CHIPOTle. The miRNAs positions were predicted by Ensembl. The definitive and putative miRNATSSs were inferred from http://mirstart.mbc.nctu.edu.tw/browse.php. The qRT-PCR data are representative of three healthy donors. The arrow points in the direction of gene transcription. R.E, relative expression; PTSS, putative transcription start site; DTSS, definitive transcription start site. *, P<0.05; **, P<0.01.

LPS is a potent inducer of DC maturation that promotes surface expression of costimulatory and antigen-presenting molecules, whereas TGF-β attenuates many of these processes [36]. We observed that H3K4me3 and H3K27me3 marks in TGF-β-conditioned DCs were very different from those in control moDCs or LPS-conditioned moDCs. The H3K4me3 peaks were significantly stronger in TGF-β-conditioned moDCs than in LPS-conditioned moDCs (Fig. S3), implying that the active genes in TGF-β-conditioned moDCs were modified by H3K4me3. Indeed, at least 7 of 23 TGF-β-upregulated miRNAs (30%), including miR-27a, miR-142 and miR-21, showed increased H3K4me3 accumulation around their TSSs (Fig. 3A and Table S4, representative samples were shown in Fig. 3B). 15 of 86 TGF-β-downregulated miRNAs (17.4%), including miR-9-1, miR-125b and miR-34a, showed increased H3K27me3 enrichment (Fig. 3C and Table S4, representative samples were shown in Fig. 3 D). Indeed, as H3K4me3 was enriched in TGF-β-conditioned moDCs, the levels of miR-27a, miR-142 and miR-21 became higher than those in the control moDCs (P<0.01), whereas H3K27me3 enrichment reduced the expression of miR-9-1, miR-125b-1 and miR-34a to below that of the control (P<0.01) (Fig. 3E). Thus, in TGF-β-associated moDCs, miRNA upregulation is associated with H3K4me3 enrichment, while H3K27me3 enrichment appears to downregulate miRNA expression.

### LPS-associated miRNAs are regulated by H3K4me3 redistribution around their transcription start sites

**Figure 4:**
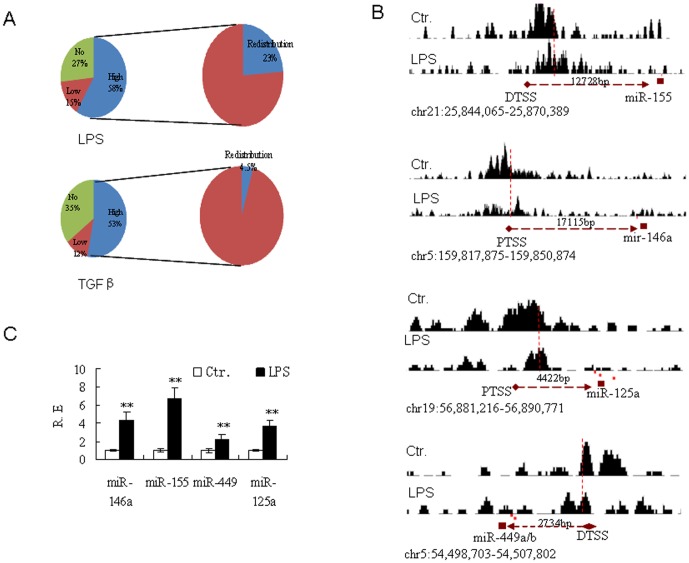
H3K4me3 redistribution from TSS to miRNA-coding region affects miRNA expression in LPS-conditioned moDCs. (**A**) Proportion of H3K4me3 redistribution around the TSSs in the LPS- (LPS) or TGF-β(TGFβ)-upregulated miRNA with H3K4me3 modification. (**B**) H3K4me3 redistribution around the TSSs in the LPS-upregulated miRNA with H3K4me3 modification. For ChIP-Seq analysis, the MoDCs were exposed to LPS (100 ng/ml) for 24 hrs. The gene structure was downloaded from the UCSC Genome Brower. Enriched regions were found in the UCSC Genome Browser (http://genome.ucsc.edu.). Histone modification peaks were detected by CHIPOTle. The miRNA positions were predicted by Ensembl. The definitive and putative miRNATSSs were inferred from http://mirstart.mbc.nctu.edu.tw/browse.php. (**C**) qRT-PCR analysis of microRNA in LPS-conditioned DCs. moDCs were exposed to LPS (100 ng/ml) for 24 hrs, and the total RNAs was extracted from LPS-conditioned DCs. R.E, relative expression. The qRT-PCR data were representative of three healthy donors. The arrow points in the direction of gene transcription. *, P<0.05; **, P<0.01.

We also investigated H3K4me3 and H3K27me3 modification in LPS-conditioned moDCs. Similar to TGF-β-conditioned moDCs, some miRNAs (such as miR-196a-1, miR-196a-2, miR-150 and miR-445) were downregulated by H3K27me3 enrichment in LPS-conditioned moDCs (unshown). However,although 126 of 172 LPS-upregulated miRNAs (73%) were modified by H3K4me3, the expression of other LPS-upregulated miRNAs, such as miR-155, miR-146a, miR-21, miR-23b, miR29, miR-34a, miR-99b and miR-148/152, was unrelated to H3K4me3 enrichment at the TSS of the promoter region (Fig. 3A and Table S5). As an alteration, the miRNAs of LPS-conditioned moDCs exhibited clear evidence of H3K4me3 redistribution around the TSS (namely, translocation from the TSS to the miRNA-coding region). At least 29 of the 126 miRNAs with H3K4me3 modification (23%) showed marked redistribution around their promoter region TSSs (Fig. 4A and Table S5, representative samples were shown in Fig. 4B), whereas only one H3K4me3 redistribution in 22 TGF-βupregulated miRNAs(4.5%) with H3K4me3 modification was found in TGF-β-conditioned moDCs (Fig. 4A and Table S5). These miRNAs were indeed markedly upregulated following exposure to LPS, relative to the control (P<0.01) (Fig. 4C). Thus, H3K4me3 redistribution may be involved in miRNA regulation in LPS-conditioned moDCs.

### H3K4me3 redistribution is regulated by LPS-mediated epigenetic factors and LPS-activated NF-κB

**Figure 5:**
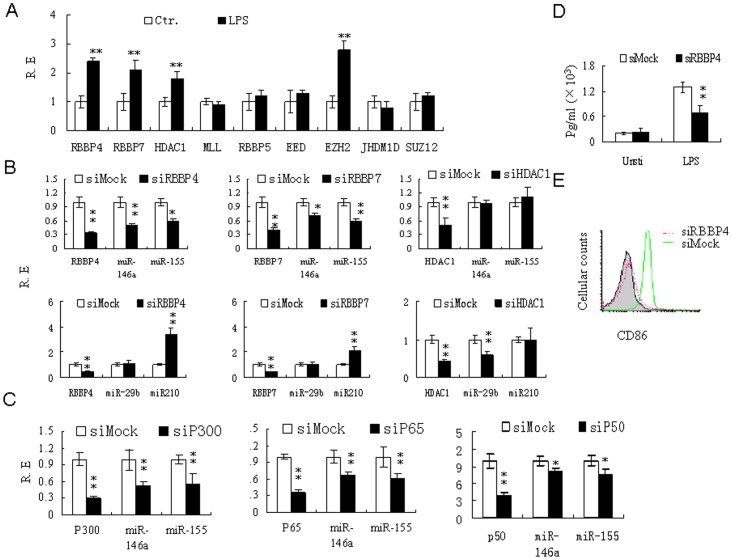
LPS associated factors affects the expression of miRNAs with H3K4me3 redistribution. (**A**) qRT-PCR analyses of epigenetic factors RBBP4, RBBP7, HDAC1, MLL, RBBP5, EED, EZH2, JHM1D and SUZ12. Total RNA was extracted from moDCs following exposure to LPS (100 ng/ml) for 24 hrs and the expressions of RBBP4, RBBP7, HDAC1, MLL, RBBP5, EED, EZH2, JHM1D and SUZ12 were analyzed by qRT-PCR. (**B**) qRT-PCR analyses of miR-146a, miR-155, miR-29 and miR-210 in siRNA-transfected moDCs. MoDCs were transfected with siRNAs against RBBP4 (siRBBP4), RBBP7 (siRBBP7), HDAC1 (siHDAC1) or with control siRNA (siMock). After 48 hrs, total RNAs were extracted and analyzed by qRT-PCR. (**C**) qRT-PCR analyses of miR-146a and miR-155 in siRNA-transfected moDCs. moDCs were respectively transfected with siRNAs against p300 (siP300), p65 (sip65), p50 (siP50), or with control siRNA (siMock). After 48 hrs, total RNA was extracted and analyzed by qRT-PCR. (**D**) TNFα ELISA analyses of supernatants from siRBBP4 transfected moDCs. The supernatants were collected from siRBBP4 transfected moDCs and the TNFα concentrations were analyzed by ELISA (R&D, USA). (**E**) Flow cytometry analysis of CD86. siRBBP4 transfected moDCs were stained with anti-CD86 (IT2.2) and analyzed by flow cytometry. Grey, isotypic control. *, P<0.05; **, P<0.01.

**Figure 6:**
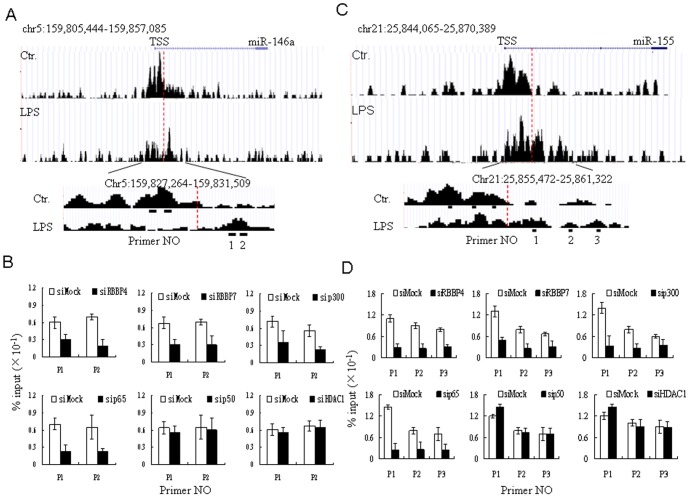
LPS activated NF-κB is involved in H3K4me3 redistribution in LPS-conditioned moDCs. (**A** and **C**) The positions of primers used in ChIP-PCR. H3K4me3 modifications at the TSS of miR-146a (**A**) and miR-155 (**C**). Significant histone modification peaks were identified by CHIPOTle. The positions of miRNAs were predicted by Ensembl. Primer positions are underlined. Number, primer No; TSS, transcription start site. (**B** and **D**) ChIP-PCR analyses of H3K4me3 enrichment around the TSSs of miR-146a and miR-155. MoDCs were transfected with siRBBP4, siRBBP7, sip300, sip65, siP50, siHDAC1 or control mock siRNA (siMock). ChIP was performed using anti-H3K4me3 antibody and H3K4me3 enrichments were analyzed by qRT-PCR. Enrichments in specific genomic regions were assessed by ChIP assay. The primer sets are listed in Table S1.

We then explored the mechanism of H3K4me3 redistribution in LPS-conditioned moDCs. RBBP4 and RBBP7 may act as histone-binding proteins and components of protein complexes involved in chromatin remodeling [37], [38]. Interestingly, the expression of these epigenetic factors is related to LPS stimulation. As shown in Fig. 5A, LPS strongly upregulated RBBP4 and RBBP7 expression, and promoted the expression of other epigenetic factors such as HDAC1 and EZH2 (Fig. 5A). Importantly, silencing RBBP4 or RBBP7 reduced miR-146a and miR-155 expression (relative to the control; P<0.01) (Fig. 5B), suggesting that LPS regulates miRNA expression via epigenetic factors. Indeed, ChIP using antibodies for H3K4me3, followed by qRT-PCR with primers flanking the H3K4me3-binding sites on the miR-155 and miR-146a promoters, showed that silencing RBBP4 and RBBP7 inhibited the redistribution of H3K4me3 from the TSS to the miRNA-coding region in the promoter regions of miR-146a and miR-155 in LPS-treated DCs (Fig. 6). Conversely, miRNAs that were not highly modified by H3K4me3 redistribution (such as miR-29b and miR-210) were not downregulated by silencing RBBP4 and RBBP7 (the results of silencing RBBP4 are shown in Fig. 5B). Silencing RBBP4 also affected TNF-α production and expression of the co-stimulatory molecule CD86 (Fig. 5D and E), which is regulated by miR-155 [15], [31].

Previous studies have suggested that CBP/p300 remodels chromatin and activates transcription via histone acetylation [39]. Here, we found that silencing CBP/p300 also interrupted the redistribution of H3K4me3 in the promoter regions of miR-155 and miR-146a (Fig. 6B and D). Importantly, this interruption significantly reduced miR-155 and miR-146a expression relative to the control (P<0.01) (Fig. 5C). Since NF-κB must interact with the co-activator CBP/p300 during enhanceosome formation [40], we next determined the effects of LPS-mediated NF-κB/p65 and p50 on H3K4me3 redistribution and on miR-155 and miR-146a expression. While p65 silencing markedly inhibited H3K4me3 redistribution from the TSS to the miRNA-coding region, NF-κB/p50 silencing exerted little effect (Fig. 6B and D). This finding reflects the different functions of p50 and p65. Generally, p50 negatively regulates inflammation by recruiting the transcriptional repressor HDAC1 [29], [41]. Similarly, we found that H3K4me3 redistribution was little affected by HDAC1silencing (Fig. 6B and D). However, miR-146a and miR-155 expression was downregulated by suppressing p50 but not by suppressing HDAC1 (P<0.05) (Fig. 5B and C), indicating that p50 plays other roles in miRNA expression.

Collectively, our results show that H3K4me3 redistribution is regulated not only by LPS-mediated RBBP4 and RBBP7 but also by LPS-mediated activation of NF-κB transcription factors.

## Discussion

DCs are extremely versatile antigen-presenting cells involved in initiating both innate and adaptive immunity and in self-tolerance maintenance. These diverse and almost contradictory functions depend largely on the plasticity of DCs, which allows them to undergo complete genetic reprogramming in response to different stimuli [8], [9]. Evidence exists that miRNAs may regulate the differentiation and function of moDCs [31], [15]. However, the mechanism for controlling miRNA expression remains obscure, especially when DCs are exposed to different environments. Here we demonstrated that miRNA expression might be regulated by H3K4me3 and H3K27me3 modification around the miRNA TSSs in TGF-β-conditioned and LPS-conditioned moDCs. H3K4me3 enrichments are associated with active miRNAs, whereas repressive miRNAs are related to H3K27me3 modification. H3K4me3 methylation and enrichment requires the MLL/RBBP5 complex, whereas EZH2 and EED mediate the methylation of H3K27me3 [3]. Indeed, we found that silencing MLL or RBBP5 inhibits both H3K4me3enrichment and miRNA expression. Conversely, silencing EZH2 or EED upregulates the expression of moDC-associated miRNAs. Previously, EED, EZH2 and SUZ12 have been shown to form repressive complexes [19], [20] that control ES cell self-renewal through histone methylation and acetylation [42]. In EED-deficient ES cells, H3K27me3 accumulation is absent, and multiple differentiation-associated genes are upregulated [42].

Notably, LPS-mediated miRNAs are often associated with the redistribution of H3K4me3 from the transcription start site to miRNA-coding region. Silencing of RBBP4 and RBBP7, which are upregulated in LPS-conditioned moDCs, reduces LPS-mediated miR-146a and miR-155 expression. Both RBBP4 and RBBP7 are histone-binding proteins. More specifically, they are components of protein complexes involved in histone deacetylation and chromatin remodeling [37], [38]. Importantly, RBBP4 interacts with the p300 complexes (CBP)-binding protein and phosphorylated p300 (CBP) [43], enabling interaction with core histones and mononucleosomes. Such binding increases the histone acetyltransferase activity of p300 and p300-mediated transcription [43]. Here, we reconfirm the critical role of p300-mediated transcription by observing the effect of p300 silencing on miR-146a and miR-155 expression. Since certain miRNAs (such as miR-146a and miR-155) are upregulated by LPS in DCs, LPS-mediated redistribution of H3K4me3 may favor the formation of preinitiation complexes, which are necessary for gene expression.

Chromatin remodeling is modulated by multiple signaling pathways. Our results suggest that the LPS-mediated NF-κB signaling pathway, which plays a critical role in the inflammatory response [44], is involved in H3K4me3 redistribution. Indeed, silencing of the NF-κB subunit p65 affected not only miR-146a and miR-155 expression but also the redistribution of H3K4me3 from the TSS to the miRNA-coding region. The p65 protein is a key active component of NF-κB. Phosphorylated p65 associates strongly with CBP/p300 and poorly with HDAC-1 [43], and may thereby induce gene expression. However, silencing the active component of NF-κB (p50) produces no similar effects on miRNA expression and H3K4me3 modification, consistent with the distinct roles of different forms of NF-κB in the inflammatory response [44]. In addition, the recruitment of HDAC-1 to inflammatory genes may be part of a widespread mechanism that explains the properties of p50 [43], [45]; for instance, the NF-κB p50:p50:HDAC1 repressor complex mediates the transcriptional inhibition of multiple pro-inflammatory genes [45]. The p65 subunit of NF-κB may also interact with histone deacetylase corepressors such as HDAC1 to negatively regulate gene expression [46]. Since HDAC1 silencing exerts no significant effect on either miR-146a and miR-155 expression or redistribution of H3K4me3, it appears that LPS-mediated NF-κB p65 and NF-κB p50 do not form repressor complexes with HDAC1.

**Figure 7:**
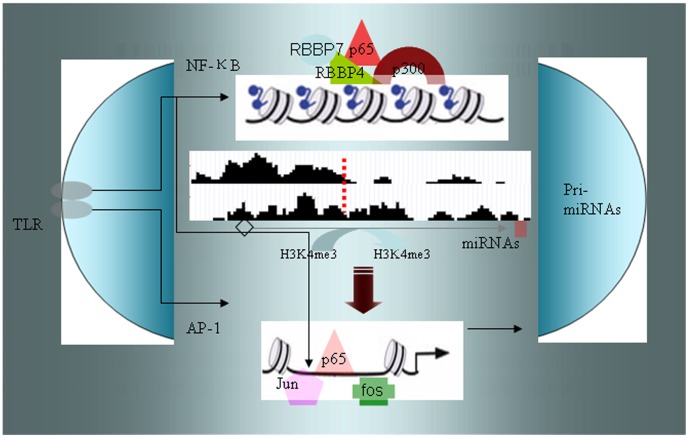
Regulation of miRNA expression in LPS-conditioned moDCs. When moDCs are exposed to LPS, activated NF-κB p65 induces H3K4me3 redistribution from the TSS to miRNA-coding region by interacting with p300 and RBBP4/RBBP7. Meanwhile, activated NF-κB transcription factors (such as p65, p52, p50, C-rel and CrelB) bind to promoter regions and initiate miRNA expression. AP-1 components Jun and c-fos, which are activated by LPS, also bind with the miR-146a and miR-155 promoters to induce miR-155 and miR-146a gene expression.

Combining our results with previous findings [47], we suggest a mechanism by which miRNAs in moDCs could be rapidly and effectively regulated in the presence of LPS (Fig. 7). Activated NF-κB p65 induces H3K4me3 modification and redistribution by interacting with p300 and RBBP4/RBBP7. This interaction enables activated NF-κB transcription factors (such as p65, p52, p50, C-rel, and CrelB) to bind to promoter regions and thereby initiate miRNA expression.

## Conclusions

The expression of miRNAs in moDCs, TGF-conditioned moDCs and LPS-conditioned moDCs may be controlled by H3K4me3 and H3K27me3 modification. H3K4me3 redistribution from the TSS to the miRNA-coding region plays a critical role in LPS-mediated expression of miRNAs. Thus, in different environments, H3K4m3 and H3K27me3 modifications and their redistribution around the TSSs of miRNAs largely regulate the differentiation and function of DCs.

## Supporting Information

Figure S1 **Silencing RBBP5 affects the expression of multiple miRNAs, the production of cytokines and the phenotype of moDCs.** (**A**) qRT-PCR anlysis of miRNAs in moDCs transfected with RBBP5 siRNA. MoDCs were transfected with RBBP5 siRNA (siRBBP5, 100 nM/10^6^). After 24 hrs, total RNA was extracted, and the expression of miRNAs was analyzed by qRT-PCR. (**B**) TNFα ELISA analysis of supernatants in siMLL (MLL siRNA)-and siRBBP5 (RBBP5 siRNA)-transfected moDCs. The supernatants were collected from siMLL- and siRBBP5- transfected moDCs after transfection for 24 hrs in the presence of LPS (100 ng/ml) or TGF-β (10 ng/ml). The TNFα concentration was analyzed by ELISA kit (R&D, USA). (**C**) CD86 flow cytometry analysis. siMLL- and siRBBP5- transfected moDCs were stained by anti-CD86 (IT2.2) after transfection for 48 hrs and then analyzed by flow cytometry. R.E, relative expression. The data from qRT-PCR are one representative of three different healthy donors. The arrow represents the direction of gene transcription.
(TIF)

Figure S2 **Analysis of miRNA expression in moDC, LPS-conditioned moDC and TGF-β-conditioned DC.** (**A**) Comparison of miRNA expression between moDCs and LPS-conditioned moDCs; (**B**) Comparison of miRNA expression between moDCs and TGF-β-conditioned DC. MoDCs were generated and visualized with anti-CD14, anti-CD83, anti-CD11c, anti-CD86, anti-CD80, anti-CD40 and anti-CD11b. The generated moDCs were exposed to TGF-β (10 ng/ml) or LPS (100 ng/ml) for 24 hrs and then expression levels of miRNAs in moDCs, LPS-conditioned moDCs and TGF-β-conditioned moDCs were analyzed using Exiqon microRNA arrays (Denmark) according to the protocol described in materials and methods.
(TIF)

Figure S3 **Enrichment of H3K4me3 on the chromatin is markedly decreased in LPS-conditioned DCs compared to unconditioned moDCs.** The generated moDCs were exposed to LPS (100 ng/ml) or TGF-β (10 ng/ml) for 48 hrs and then analyzed by ChIP-Seq. Enriched regions were found in the UCSC Genome Browser (http://genome.ucsc.edu.). A screen shot from the UCSC Genome Browser shows the distribution of histone modifications. Histone modifications peaks were identified by CHIPOTle. The positions of miRNAs predicted by Ensembl are shown.
(TIF)

Table S1 Oligos used in this study.
(DOC)

Table S2 miRNA array of moDC, LPS-conditioned moDCs and TGF-βconditioned moDCs.
(XLS)

Table S3 moDC-associated miRNAs with H3K4me3, H3K27me3 or H3K4me3 and H3K27me3 modification.
(XLS)

Table S4 TGFβor LPS-associated miRNAs with H3K4me3 or H3K27me3 modifications.
(XLS)

Table S5 LPS and TGF-βupregulated miRNAs with H3K4me3 redistribution.
(XLS)
