# Supplementary figures and images for: Dendritic Cell-Associated miRNAs Are Modulated via Chromatin Remodeling in Response to Different Environments
Source: PLoS One. 2014 Apr 3;9(4):e90231. doi: 10.1371/journal.pone.0090231 (PMC3974670; doi:10.1371/journal.pone.0090231)

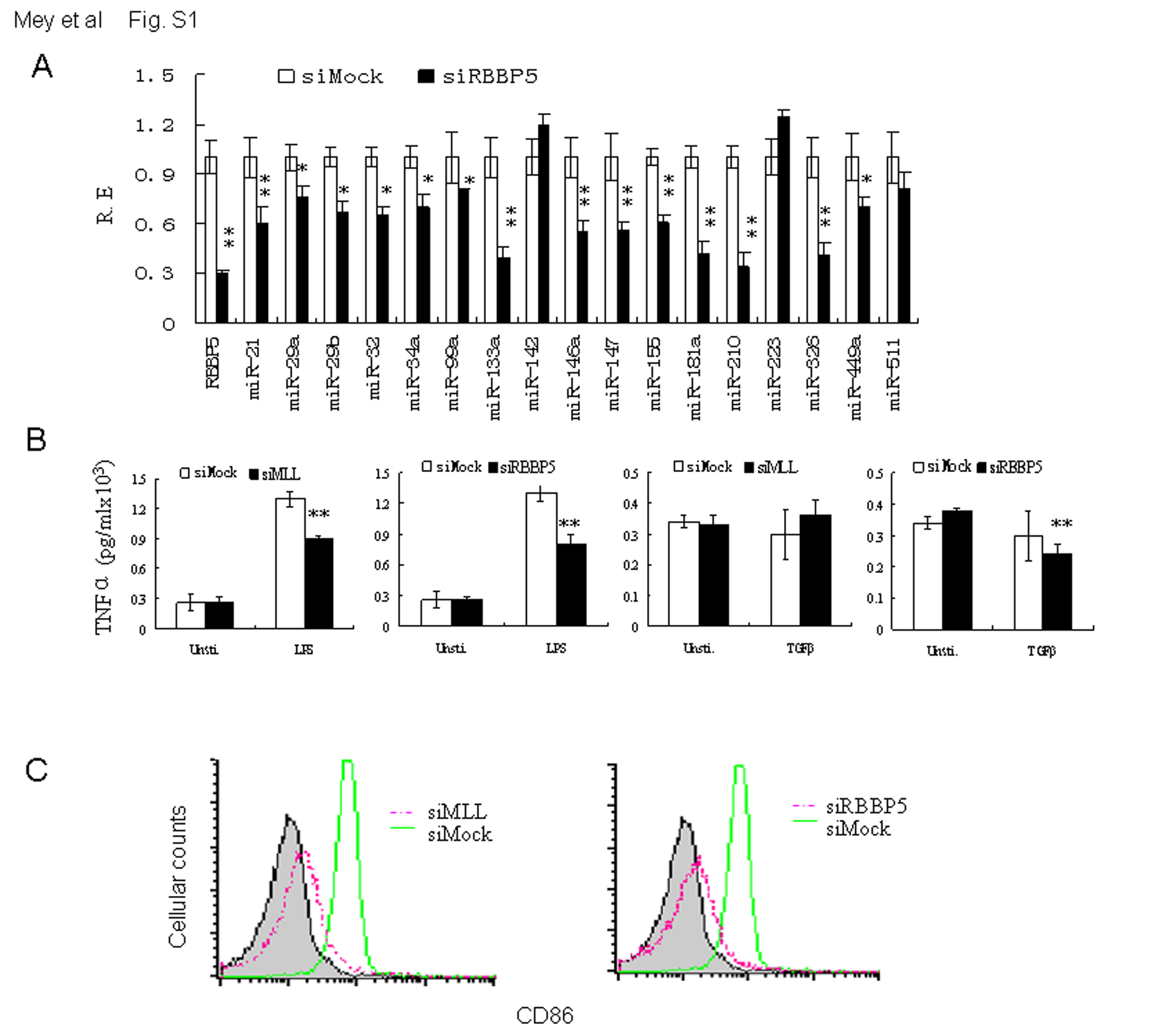

Supplement: Figure S1 — Silencing RBBP5 affects the expression of multiple miRNAs, the production of cytokines and the phenotype of moDCs. (A) qRT-PCR anlysis of miRNAs in moDCs transfected with RBBP5 siRNA. MoDCs were transfected with RBBP5 siRNA (siRBBP5, 100 nM/106). After 24 hrs, total RNA was extracted, and the expression of miRNAs was analyzed by qRT-PCR. (B) TNFα ELISA analysis of supernatants in siMLL (MLL siRNA)-and siRBBP5 (RBBP5 siRNA)-transfected moDCs. The supernatants were collected from siMLL- and siRBBP5- transfected moDCs after transfection for 24 hrs in the presence of LPS (100 ng/ml) or TGF-β (10 ng/ml). The TNFα concentration was analyzed by ELISA kit (R&D, USA). (C) CD86 flow cytometry analysis. siMLL- and siRBBP5- transfected moDCs were stained by anti-CD86 (IT2.2) after transfection for 48 hrs and then analyzed by flow cytometry. R.E, relative expression. The data from qRT-PCR are one representative of three different healthy donors. The arrow represents the direction of gene transcription. (TIF) [file pone.0090231.s001.tif]

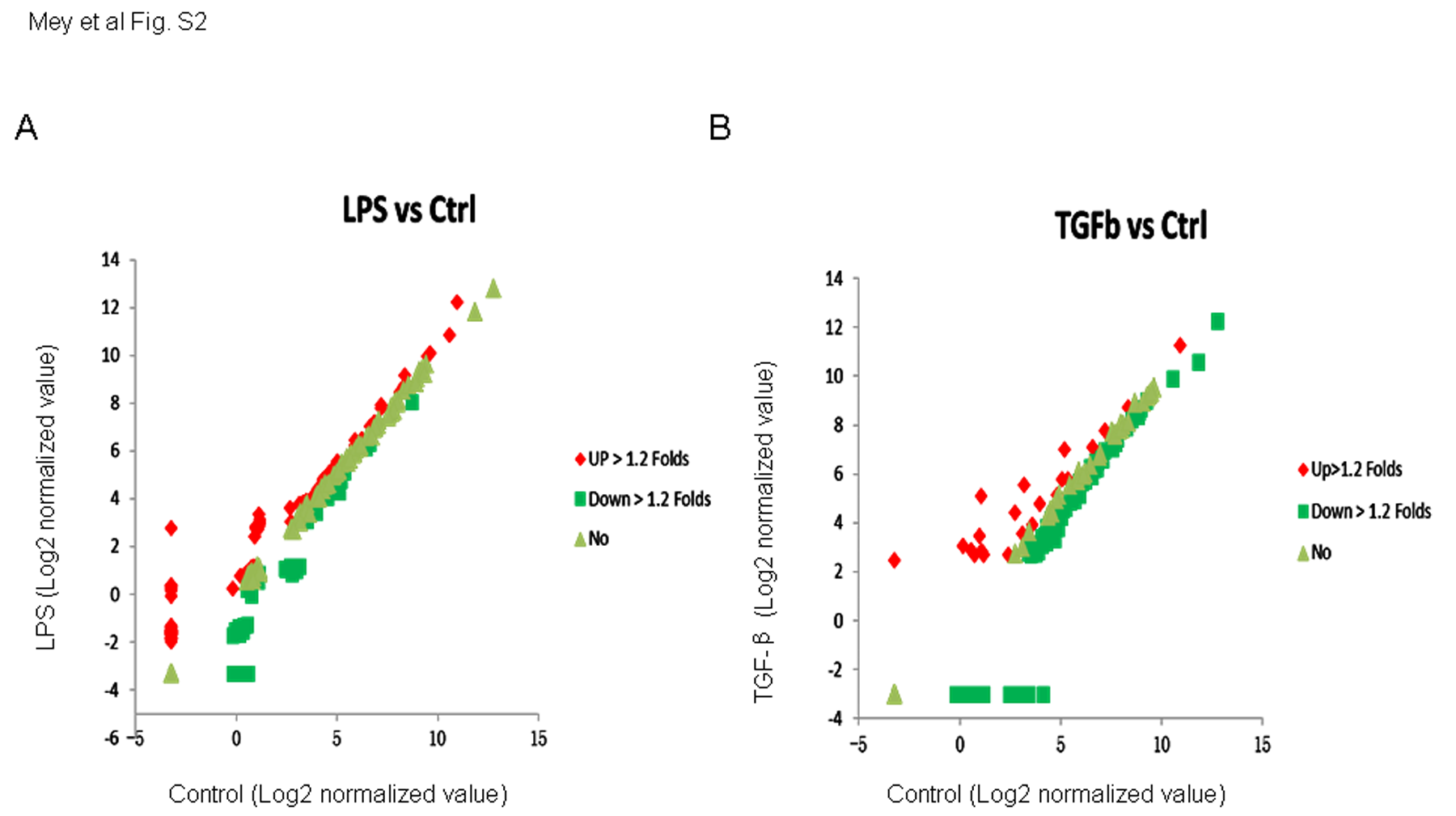

Supplement: Figure S2 — Analysis of miRNA expression in moDC, LPS-conditioned moDC and TGF-β-conditioned DC. (A) Comparison of miRNA expression between moDCs and LPS-conditioned moDCs; (B) Comparison of miRNA expression between moDCs and TGF-β-conditioned DC. MoDCs were generated and visualized with anti-CD14, anti-CD83, anti-CD11c, anti-CD86, anti-CD80, anti-CD40 and anti-CD11b. The generated moDCs were exposed to TGF-β (10 ng/ml) or LPS (100 ng/ml) for 24 hrs and then expression levels of miRNAs in moDCs, LPS-conditioned moDCs and TGF-β-conditioned moDCs were analyzed using Exiqon microRNA arrays (Denmark) according to the protocol described in materials and methods. (TIF) [file pone.0090231.s002.tif]

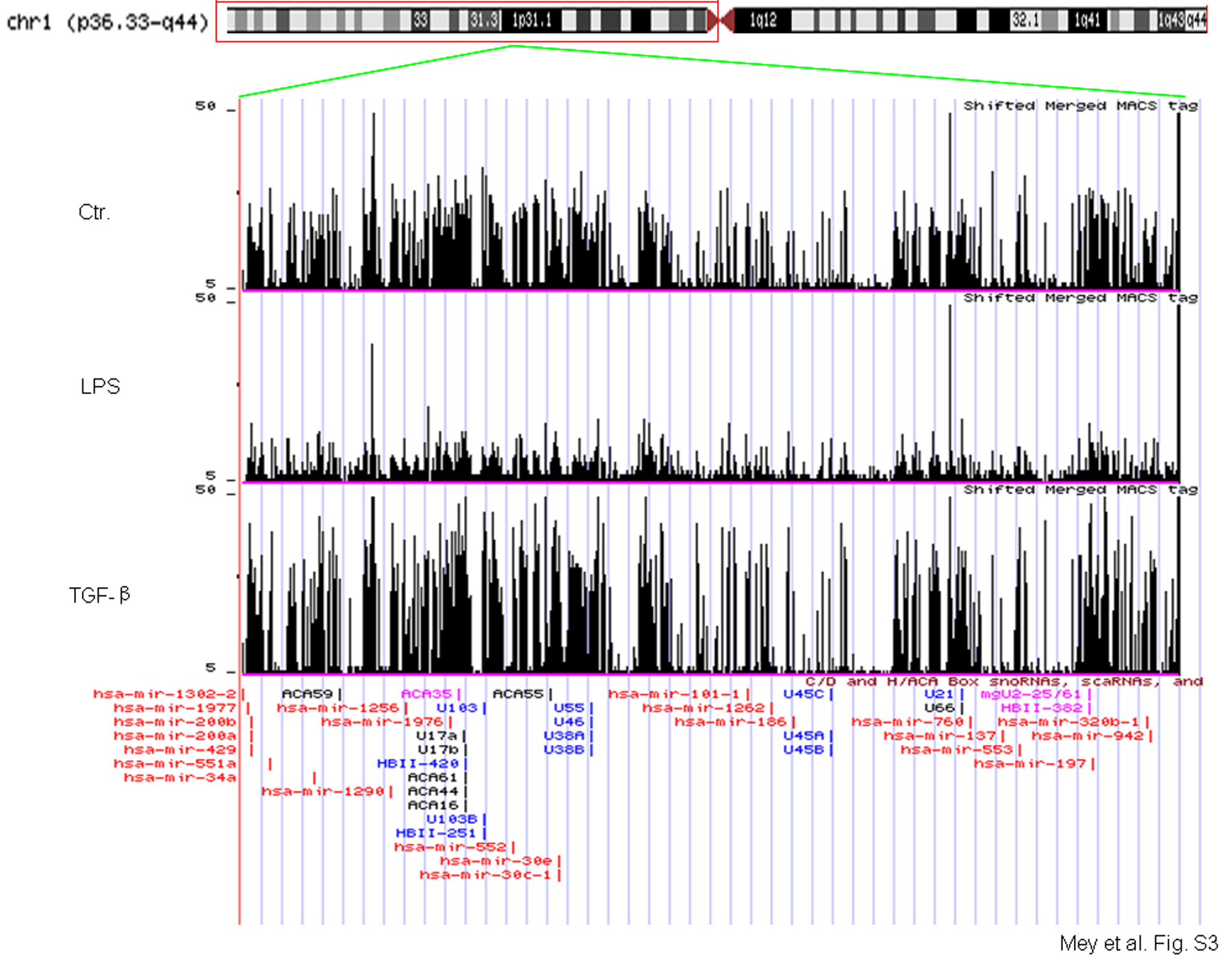

Supplement: Figure S3 — Enrichment of H3K4me3 on the chromatin is markedly decreased in LPS-conditioned DCs compared to unconditioned moDCs. The generated moDCs were exposed to LPS (100 ng/ml) or TGF-β (10 ng/ml) for 48 hrs and then analyzed by ChIP-Seq. Enriched regions were found in the UCSC Genome Browser (http://genome.ucsc.edu.). A screen shot from the UCSC Genome Browser shows the distribution of histone modifications. Histone modifications peaks were identified by CHIPOTle. The positions of miRNAs predicted by Ensembl are shown. (TIF) [file pone.0090231.s003.tif]
