# Supplementary material for: Dendritic Cell-Associated miRNAs Are Modulated via Chromatin Remodeling in Response to Different Environments
Source: PLoS One. 2014 Apr 3;9(4):e90231. doi: 10.1371/journal.pone.0090231 (PMC3974670; doi:10.1371/journal.pone.0090231)
Supplement: Table S1 — Oligos used in this study. (DOC) [file pone.0090231.s004.doc]

**Table S1.** Oligos used in this study.

A. Primers for mature miRNA quantitation.

| Oligo name | Stem loop RT primer (1) and qPT-PCR Foward-primer (2) |
| --- | --- |
| hsa-miR-146a | GTCGTATCCAGTGCAGGGTCCGAGGTATTCGCACTGGATACGACaaccca (1)  GCCGTGAGAACTGAATTCCA (2) |
| hsa-miR-155 | GTCGTATCCAGTGCAGGGTCCGAGGTATTCGCACTGGATACGACacccct(1)  CGGCTTAATGCTAATCGTGAT(2) |
| hsa-miR-21 | GTCGTATCCAGTGCAGGGTCCGAGGTATTCGCACTGGATACGAC tcaaca (1)  CGGCTAGCTTATCAGACTGA (2) |
| hsa-miR-29a | GTCGTATCCAGTGCAGGGTCCGAGGTATTCGCACTGGATACGAC taaccg (1)  CGGCTAGCACCATCTGAAAT (2) |
| hsa-miR-29b | GTCGTATCCAGTGCAGGGTCCGAGGTATTCGCACTGGATACGACaacact (1)  CGCGTAGCACCATTTGAAATC (2) |
| hsa-miR-32 | GTCGTATCCAGTGCAGGGTCCGAGGTATTCGCACTGGATACGAC tgcaac (1)  GCCGCTATTGCACATTACTAA (2) |
| hsa-miR-34a | GTCGTATCCAGTGCAGGGTCCGAGGTATTCGCACTGGATACGACacaacc (1)  ATCGTGGCAGTGTCTTAGCT |
| hsa-miR-99a | GTCGTATCCAGTGCAGGGTCCGAGGTATTCGCACTGGATACGACcacaag (1)  ATGCAACCCGTAGATCCGAT (2) |
| hsa-miR-133a | GTCGTATCCAGTGCAGGGTCCGAGGTATTCGCACTGGATACGACcagctg (1)  CGACTTTGGTCCCCTTCAAC (2) |
| hsa-miR-142-3p | GTCGTATCCAGTGCAGGGTCCGAGGTATTCGCACTGGATACGAC tccata (1)  GCCGTGTAGTGTTTCCTACTT (2) |
| hsa-miR-147a | GTCGTATCCAGTGCAGGGTCCGAGGTATTCGCACTGGATACGACgcagaa (1)  CCGAGTGTGTGGAAATGC (2) |
| hsa-miR-181a | GTCGTATCCAGTGCAGGGTCCGAGGTATTCGCACTGGATACGACactcac (1)  ATTCAACATTCAACGCTGTCG (2) |
| hsa-miR-210 | GTCGTATCCAGTGCAGGGTCCGAGGTATTCGCACTGGATACGAC tcagcc (1)  ATTACTGTGCGTGTGACAGC (2) |
| hsa-miR-223 | GTCGTATCCAGTGCAGGGTCCGAGGTATTCGCACTGGATACGACaactca (1)  GCAACGTGTATTTGACAAGC (2) |
| hsa-miR-326 | GTCGTATCCAGTGCAGGGTCCGAGGTATTCGCACTGGATACGACctggag (1)  ATCGCCTCTGGGCCCTTC (2) |
| hsa-miR-449a | GTCGTATCCAGTGCAGGGTCCGAGGTATTCGCACTGGATACGACaccagc (1)  CGGCTGGCAGTGTATTGTTA (2) |
| hsa-miR-511 | GTCGTATCCAGTGCAGGGTCCGAGGTATTCGCACTGGATACGAC tgactg (1)  GCGAGTGTCTTTTGCTCTG (2) |
| hsa-miR-27a | GTCGTATCCAGTGCAGGGTCCGAGGTATTCGCACTGGATACGACgcggaa (1)  CGCATTCACAGTGGCTAAG(2) |
| hsa-miR-9 | GTCGTATCCAGTGCAGGGTCCGAGGTATTCGCACTGGATACGAC tcatac (1)  GCGCTCTTTGGTTATCTAGCT(2) |
| hsa-miR-125a | GTCGTATCCAGTGCAGGGTCCGAGGTATTCGCACTGGATACGAC tcacag (1)  ATTAGC TCCCTGAGACCCT(2) |
| hsa-miR-125b | GTCGTATCCAGTGCAGGGTCCGAGGTATTCGCACTGGATACGAC tcacaa (1)  AGTATCCCTGAGACCCTAAC(2) |
| hsa-miR-196a | GTCGTATCCAGTGCAGGGTCCGAGGTATTCGCACTGGATACGACcccaac (1)  GCGATAGGTAGTTTCATGTT (2) |
| hsa-miR-150 | GTCGTATCCAGTGCAGGGTCCGAGGTATTCGCACTGGATACGACcactgg (1)  AGCGTCTCCCAACCCTTGTA (2) |
| hsa-miR-455 | GTCGTATCCAGTGCAGGGTCCGAGGTATTCGCACTGGATACGACcgatgt (1)  AGCGTATGTGCCTTTGGACT (2) |
| U6 | CTCGCTTCGGCAGCACA (forward)  AACGCTTCACGAATTTGCGT (reverse) |
| Universal reverse primer | GTGCAGGGTCCGAGGT |

B. siRNA used in this study.

| siRNA name | Sense (5’-3’) |
| --- | --- |
| Mll | AAGAAGUCAGAGUGCGAAGUC |
| RBBP5 | GAGCCGAGAUGGUCAUAAAdTdT |
| EZH2 | AAGAGGUUCAGACGAGCUGAUdTdT |
| EED | AAGCACUAUGUUGGCCAUGGAdTdT |
| RBBP4 | GAUACUCGUUCAAACAAUAdTdT |
| RBBP7 | GACGCAAGAUGGCGAGUAAdTdT |
| P300 | GCACAAAUGUCUAGUUCUUdTdT |
| P65 | GAUUGAGGAGAAACGUAAAdTdT |
| P50 | GUCACUCUAACGUAUGCAAdTdT |
| HDAC1 | CCCGGAGGAAAGUCUGUUAdTdT |

C. Primers for gene quantitation.

| Gene name | Forward (5'-3') &Reverse (5'-3') |
| --- | --- |
| MLL | CAGTGTGCGTTATGTTTGACTTATG &TGCACATTCTTTAGTGATCCGTC |
| RBBP5 | ACCGGATGCAGTCCAAACC& TGCTCCTCCTGCTTTTGATCC |
| EZH2 | TTGTTGGCGGAAGCGTGTAAAATC &TCCCTAGTCCCGCGCAATGAGC |
| EED | TTGTGTGCTGGAAACCTGGC&GCCAACTTGATTGCCCAATG |
| RBBP4 | TTGATGCGTCACACTACGACAG&CAAGTCTGGGTTGCACTCTCC |
| RBBP7 | TTGAGTGGACATCTCCTAAGTG&CCTGGTGTCCCATATCATAAGT |
| P300 | CCGAAGAAGAGATTTCCTGAG&GTCGTGCTCCAAGTCAAATAG |
| P65 | CCATCAAGATCAATGGCTACAC&GCTTCTTCACACACTGGATTC |
| P50 | GTGCAGAGGAAACGTCAGAA &GTGGGAAGCTATACCCTGGA |
| HDAC1 | CTTTAACCTGCCTATGCTGATG&CTCATTCGTGTTCTGGTTAGTC |

D. primers for CHIP-PCR used in Fig. 2.

| Oligo name | Forward (5'-3') &Reverse (5'-3') |
| --- | --- |
| miR-146a  P0  P1  P2  P3  P4  P5 | TGAAGTGCAGGTGGTGTGGT & TCACATGAACATGCGCGGAG  TGTGCCGAGGAGGGATCTAGAAG& AAGGAAGAGCGGTCAAGCGT  ATGCCAAAGGGTGGACAGGA &ACAAAAGCCTGCTGCCTCTCA  TGGGTTTTTGGACAGAACTGGCT& ACTCCAAACAACCGGCACGA  TCGTGCCGGTTGTTTGGAGT &AGGGCCTTTGGAGAAAGTGGGA  ACTGGAGACAGAAGGCAGAGT& AGTAGCAGCAGCAGCAAGAG |
| miR-155  P0  P1  P2  P3  P4  P5  P6 | TCACCAATAAAGCCCTAATCACC& GACAACCACCTTCTTGCTATGTTC  ACACGCAATGACCCACGAGA& TGTGACTCATAACCGACCAGGC  AGATTGCGCTGGATGGATTC& ACACACTTCTCTGGCCACTCCA  AGAGGGGCAGGGTGGAAAAAGA& CCTGGCACAACAGGAGCGAATA  TGGCAGGGTTAGGTGGTGGTATC& AATCAGGGAGGCAGCAAGGAGT  ACTCCTTGCTGCCTCCCTGATT &TGGTTAGGAAGCTGGGTCCTGT  ATTGGGGCACCAAGTTGGCA& TTCTGCCGCTGCAGTTTTGG |

E. primers for CHIP-PCR used in Fig. 5.

| Oligo name | Forward (5'-3') &Reverse (5'-3') |
| --- | --- |
| miR-146a  P1  P2 | GGGACACCAGGAACAGAGAGAAG& GCACACTGGAGCAGCAAAGAG  CCTTTCCCTTTGACTTCTGTAACT&AGACTGACAGGTTTGAGACTATCT |
| miR-155  P1  P2  P3 | AGAGGGGCAGGGTGGAAAAAGA& CCTGGCACAACAGGAGCGAATA  TCGATGTTAGCAGGCTAGAGAC & CCAGTCCAAGTGATAGAGGTGC  GGGAGCCTGTGTAAGTGTTTG & TTTGGTTAAGGAGAAGGATATGC |
